# Establishment of a high-fidelity patient-derived xenograft model for cervical cancer enables the evaluation of patient’s response to conventional and novel therapies

**DOI:** 10.1186/s12967-023-04444-5

**Published:** 2023-09-09

**Authors:** Liting Liu, Min Wu, Anni Huang, Chun Gao, Yifan Yang, Hong Liu, Han Jiang, Long Yu, Yafei Huang, Hui Wang

**Affiliations:** 1 Department of Obstetrics and Gynecology, Tongji Hospital, Tongji Medical College, Huazhong University of Science and Technology, Wuhan, China; 2 Cancer Biology Research Center (Key Laboratory of the Ministry of Education), Tongji Hospital, Tongji Medical College, Huazhong University of Science and Technology, Wuhan, China; 3 Department of Pathogen Biology, School of Basic Medicine, Tongji Medical College, Huazhong University of Science and Technology, Wuhan, China; 4 Zhejiang Provincial Key Laboratory of Precision Diagnosis and Therapy for Major Gynecological Diseases, Women’s Hospital, Zhejiang University School of Medicine, Hangzhou, Zhejiang China

**Keywords:** Cervical cancer, PDX, Chemotherapy, Adoptive T-cell therapy, Neratinib

## Abstract

**Background:**

Recurrent or metastatic cervical cancer (r/m CC) often has poor prognosis owing to its limited treatment options. The development of novel therapeutic strategies has been hindered by the lack of preclinical models that accurately reflect the biological and genomic heterogeneity of cervical cancer (CC). Herein, we aimed to establish a large patient-derived xenograft (PDX) biobank for CC, evaluate the consistency of the biologic indicators between PDX and primary tumor tissues of patients, and explore its utility for assessing patient’s response to conventional and novel therapies.

**Methods:**

Sixty-nine fresh CC tumor tissues were implanted directly into immunodeficient mice to establish PDX models. The concordance of the PDX models with their corresponding primary tumors (PTs) was compared based on the clinical pathological features, protein biomarker levels, and genomic features through hematoxylin & eosin staining, immunohistochemistry, and whole exome sequencing, respectively. Moreover, the clinical information of CC patients, RNA transcriptome and immune phenotyping of primary tumors were integrated to identify the potential parameters that could affect the success of xenograft engraftment. Subsequently, PDX model was evaluated for its capacity to mirror patient’s response to chemotherapy. Finally, PDX model and PDX-derived organoid (PDXO) were utilized to evaluate the therapeutic efficacy of neratinib and adoptive cell therapy (ACT) combination strategy for CC patients with human epidermal growth factor receptor 2 (HER2) mutation.

**Results:**

We established a PDX biobank for CC with a success rate of 63.8% (44/69). The primary features of established PDX tumors, including clinicopathological features, the expression levels of protein biomarkers including Ki67, α-smooth muscle actin, and p16, and genomics, were highly consistent with their PTs. Furthermore, xenograft engraftment was likely influenced by the primary tumor size, the presence of follicular helper T cells and the expression of cell adhesion-related genes in primary tumor tissue. The CC derived PDX models were capable of recapitulating the patient’s response to chemotherapy. In a PDX model, a novel therapeutic strategy, the combination of ACT and neratinib, was shown to effectively inhibit the growth of PDX tumors derived from CC patients with HER2-mutation.

**Conclusions:**

We established by far the largest PDX biobank with a high engraftment rate for CC that preserves the histopathological and genetic characteristics of patient’s biopsy samples, recapitulates patient’s response to conventional therapy, and is capable of evaluating the efficacy of novel therapeutic modalities for CC.

**Supplementary Information:**

The online version contains supplementary material available at 10.1186/s12967-023-04444-5.

## Background

Cervical cancer (CC) is a severe public health concern, particularly in developing countries. It is the fourth most common cancer in women [1] and accounts for more than 300,000 deaths annually [2]. Although early-stage CC is mostly curable, r/m CC is always accompanied by a poor prognosis due to the lack of effective treatment modality [3, 4]. To develop and evaluate novel therapeutic strategy for advanced CC, preclinical models that are capable of accurately resembling PTs of patients should be developed and used to facilitate the application of therapeutic approaches into clinical practice [5].

Presently, cell lines and cell line-derived xenograft (CDX) models represent the most common tools in preclinical research for cancer [6]. However, individual cell line cannot reflect the heterogeneity of patient’s tumor. In addition, upon multiple passages, cell lines may undergo genetic drifts or mutations. These intrinsic defects result in the poor performance of the above-mentioned models in assessing patient’s response to a certain treatment [7]. Organoid model, originating from primary tumor and requiring limited culture, could largely preserve the genetic features of its corresponding PT and can be used for high throughput analysis [8]. However, the in vitro nature of this model prevents it from assessing the systemic safety profiles of candidate therapeutic modalities. Patient-derived xenograft (PDX) tumor, an in vivo model derived from individual patients, is considered an ideal tool for tumor research and has been used to explore tumor mechanisms and therapeutic modalities [9]. There have been multiple PDX models established for CC. However, these investigations have several caveats including the insufficient scale of established PDX, and limited parameters used for comparative studies to evaluate the consistency between primary tumor and PDX tumor, hence limiting the application of PDX model in cervical cancer treatment research [5, 10–13]. Additionally, key factors beyond patient clinical information [11, 12], such as the differentially expressed genes (DEGs) of tumor or stroma and the tumor immune microenvironment (TME), should be evaluated for their influence on the growth of PDX tumors for guidance in subsequent PDX establishment. Therefore, there is an urgent need, at least in CC, for larger and more comprehensive PDX biobanks establishment.

The prevalence of HER2 gene amplification or mutation in CC patients ranges from 4.8 to 17% [14, 15], and HER2 positivity is associated with a more advanced disease stage and worse prognosis in CC [16]. Fortunately, neratinib, an irreversible pan-HER inhibitor, has been shown effective in treating advanced-stage CC with HER2 mutation [17, 18]. However, its efficacy remains to be improved. Some indirect but compelling evidences suggest that ACT therapy with tumor-infiltrating lymphocytes (TILs) may have a synergistic effect with neratinib. First, anti-HER2-targeted therapy increased the levels of tumor-infiltrating lymphocytes (TILs) in solid tumors [19]. Second, several clinical trials have demonstrated that the higher the level of TILs infiltrated in tumors, the greater the clinical benefits of anti-HER2 agents were shown [20–22]. Third, ACT itself is a promising option for advanced-stage CC, which has been proved to be a promising therapy for CC with objective response rates (ORRs) of 44% and 50% in two clinical trials [23, 24]. Therefore, it is of great significance to explore whether TILs could be combined with neratinib to improve the therapeutic efficacy for CC patients with HER2-mutation.

In the present study, we established by far the largest panel of PDX models with a high success rate. We found that xenograft engraftment and grafting speed are influenced by the primary tumor size, the presence of follicular helper T cells and the expression of cell adhesion-related genes in primary tumor tissue. The established PDX tumors retained the genetic and histopathological characteristics of the corresponding patient biopsy samples, even after serial passage or cryopreservation. Furthermore, our PDX models faithfully recapitulated patients’ response to conventional chemotherapy. Finally, using the PDX model, we found that combinatorial therapy with ACT and neratinib could effectively inhibit the growth of PDX tumors derived from CC patients with HER2-mutation.

## Materials and methods

### Patients and tissue samples

We enrolled 69 CC patients, including those with stage IB1 to IIIC2 disease, admitted in Tongji Hospital of Huazhong University of Science and Technology between June 2018 and January 2021. All patients provided written informed consent. Fresh tissue specimens, measuring 200–1000 mm^3^ in size, were collected from surgery or biopsy and transported immediately to the laboratory while submerged in tissue storage solution (MACS, 130-100-008) on ice. The tissue samples were selected for implantation in mice, ribonucleic acid (RNA) and deoxyribonucleic acid (DNA) extraction, and flow cytometry analysis based on specific criteria. The research protocol received ethical approval from the Ethical Committee of Tongji Medical College, Huazhong University of Science and Technology.

### Animals

Female nonobese diabetic/severe combined immunodeficiency (NOD/SCID) NOD-SCID and NOD/SCID IL2Rγ^−/−^ (NCG) mice, aged five to eight weeks and weighed 18–21 g, were purchased from Nanjing Biomedical Research Institute of Nanjing University (Nanjing, China). The experimental mice were housed in isolator cages maintained under specific-pathogen-free conditions, with precisely regulated temperature and humidity, and with a standardized 12 h light/dark cycle at Tongji Medical College. All animal care and experimental procedures strictly adhered to the guidelines for ethical review of animal welfare and were granted approval by the Institutional Animal Care and Use Committee at Huazhong University of Science and Technology.

### Establishment of PDX models

The PT used for implantation measured 3–4 mm^3^ in size and was implanted subcutaneously into the flanks of NOD/SCID mice. When the tumors (P1 generation) reached 1500 mm^3^ in size, they were resected and introduced into the mice (P2 generation). The process was repeated until the P4 generation was generated. Of note, PT and PDX tumor are free of common human and animal pathogens as determined by PCR-based methodology (data not shown).

### Establishment and culture of PDXO

PDXOs were established and cultured as previously described [8]. Briefly, the cells were resuspended in basement membrane extracts (Corning, Corning, NY, USA) and cultured on a basal medium that containing growth factors such as Noggin, FGF7, B27, Y-27632, and EGF [8].

### Generation and rapid expansion of TILs

The surgical specimens were obtained and cut into 1–2 mm^2^-thick sections, which were then placed in a 24-well plate with 1 mL of culture medium consisting of 90% RPMI 1640 (Gibco) and 10% heat-inactivated human AB serum (HS). Recombinant human interleukin (IL)-2 (6000 IU/mL; GeneScript) was added, along with 1% penicillin and streptomycin. The TILs were expanded in a 75 cm^2^ cell culture flask using a standard rapid expansion protocol (REP) with irradiated (50 Gy) feeder cells (1 × 10^8^); moreover, 40 mL culture medium containing 90% REP medium (X-Vivo, Lonza), 10% HS, 3000 IU/mL IL-2 (GenScript), and 25 mM β-mercaptoethanol (Sigma) were added as supplements, along with 30 ng/mL CD3 antibody (clone: OKT3, BioLegend) and TILs (1 × 10^6^). On day 7 and every day thereafter, the cell densities were maintained at 1–2 × 10^6^ cells/mL. On days 12–14, the cells were harvested and cryopreserved in liquid nitrogen. The TILs were then injected intravenously into the mice at a dose of 100 μl (10 × 10^6^ TILs per mice).

### Cell growth assay and organoid-TIL co-culture systems

Organoid growth was assessed utilizing the standard Cell Counting Kit-8 assay (Vazyme, Jiangsu, China) following the manufacturer’s protocol.

TILs were culture in X-Vivo medium, supplemented with 25 mM β-mercaptoethanol, 1:100 penicillin/streptomycin, and human AB serum (“TIL cell medium”). Prior to co-culture, cryopreserved REP TIL was thawed in pre-warmed (37 °C) TIL cell medium and incubate for 15 min at 37 °C with 60 IU/mL DNase I (Sangon Biotech). After that, resuspend cell at 2 × 10^6^ per mL in TIL cell medium and add 150 IU/mL of IL2 and cultured overnight. Organoids are being cultured overnight with 200 ng/mL IFN-γ (PeproTech). 96-well U-bottom plates were coated with 5 μg/mL anti-CD28 (Biolegend) and kept overnight at 4 °C. On the coculture day, PDXO were dissociated to single cells with TrypLE Express and resuspended in TIL cell medium at 5 × 10^4^ cell per mL. TIL was seeded at a density of 1 × 10^6^ cells/well and stimulated with single cell organoids at a 20:1 TIL: tumor cell ratio, supplement with 150 IU/mL IL-2 and 20 μg/mL anti-PD-1. Plate 200 μL of dissociated organoid-TIL suspension per well and incubate at 4 °C for two days [25].

### CTL assay

The organoids were digested into single cell suspensions and stimulated for 24 h with 200 ng/mL of anti-interferon-α. After washing with phosphate-buffered saline (PBS), the cells were seeded at a density of 5 × 10^4^ cells per well in 96-well round-bottom plates. The target cells were cultured in duplicate with effector Rep TIL cells in 200 μl TIL cell medium at 37 °C in a CO_2_ incubator for 4–6 h at the indicated an E/T ratio of 20:1, 10:1, and 5:1. Percent specific lysis was calculated using the following formula: 100 × [(experimental release − spontaneous release)/(maximum release − spontaneous release)], following the LDH Cytotoxicity Assay Kit instructions.

### Mouse experiments

Under anesthesia, tumor fragments measuring 3–4 mm^3^ in size were grafted into flanks of NCG mice. Treatment began after the tumor reached 70 mm^3^ in size. The following drugs and dosage regimens were used. Neratinib (MedChem Express) 20 mg/kg was administered five times a week via oral gavage for 20 days. Autologous TILs were infused via tail vein injection with a dose of 10 × 10^6^ cells. IL-2 was given at a dose of 45,000 IU for 3 days initially, followed by twice weekly dosing. Cisplatin 5 mg/kg diluted in water was administered intraperitoneally twice a week. The tumor size and mouse weight were assessed at the initiation of treatment, twice a week, and at the end of treatment. The volumes were determined using the following formula: 0.52 × length (L) × width (W)^2^, where L represents the major tumor axis and W represents the minor tumor axis [26].

### DNA extraction, whole exome sequencing and data analysis

DNA was extracted from frozen tumor and normal tissue samples of patients and matched PDX tumor samples using the DNeasy Blood and Tissue Kit (Qiagen, Germantown, ML, USA). The DNA quality was determined using Qubit 3.0, while the DNA samples were clustered using the Illumina PE Cluster Kit on the cBot Cluster Generation System (Illumina, San Diego, CA, USA). The whole exome sequencing (WES) libraries were prepared from 1 μg of genomic DNA using an Agilent liquid capture system (SureSelect Human All Exon V6; Agilent, Santa Clara, CA, USA) and sequenced with the Illumina HiSeq Instrument using 180–280-bp paired-end reads generated on the Novogene platform according to the manufacturer’s instructions.

Using the BMA program, the reference human + mouse (hg 19 + mm 10) genome was used to map the sequence reads from each model [27]. The NovaSeq 6000 had a 20-fold mean read depth for PT samples that matched the PDX samples and a tenfold mean read depth for matching normal samples. Species disambiguation was performed using the software available at https://github.com/disambiguate. VarScan v.2.3 was used to identify the single nucleotide variants (SNVs) [28], and the results were annotated using ANNOVAR. The CNVkit (version 0.9.3) was utilized to detect the copy number variations (CNVs), while the segmented data were detected using Genomic Identification of Significant Targets in Cancer (GISTIC) 2.0 [29]. The CNV discordance between samples was determined by assessing log_2_ (CN ratio) CN gains and losses, and three samples were excluded from the analysis based on the criterion [30].

### RNA sequencing and differential gene analysis

RNA was extracted from the patient’s tumors using the RNA Easy Mini Kit (Qiagen, CA), following the manufacturer’s instructions. Sequencing was performed on Illumina NovaSeq 6000 with paired 150-bp reads. The StringTie software was employed to extract read counts and fragments per kilobase of transcript sequence per million base pairs sequenced. The edgeR package was used to standardize the RNA sequencing data and identify differentially expressed genes (DEGs). In our dataset, the genes with fewer than 1 count in less than 5 samples were excluded, and genes with at least two-fold change with a *p*-value of < 0.05 were considered to be DEGs. Gene set enrichment analysis was performed using the GSEA software (V4.1.0). Meanwhile, the ClusterProfiler package was used to perform Kyoto Encyclopedia of Genes and Genomes (KEGG) enrichment analysis.

### Immunohistochemistry

PDX and fresh tumor specimens were fixed in 4% formalin, embedded in paraffin, and stained with hematoxylin and eosin (H&E) or immunohistochemistry (IHC). The tissue was then dehydrated, and antigen retrieval with AR6 (sodium citrate) or AR9 (EDTA) was optimized. The slides were then observed and photographed under an Olympus BX53 light microscope. The epithelial and stromal components of the tumor were demarcated using the inForm software v2.6 (Akoya)^®^, and the IHC markers were quantified.

### Multiplex IHC staining

Briefly, the slides were dewaxed and rehydrated, and antigen retrieval was performed. The endogenous peroxidase and Fc receptor were blocked as described in the IHC protocols. The samples were stained with rabbit monoclonal anti-CDKN2A (1:1,000. Abcam), rabbit monoclonal anti-CD4 (1:5, SP8, MXB), and rabbit monoclonal anti-CD8 (1:5, SP16, MXB). The Opal 7-Color Manual IHC Kit (Akoya Biosciences, DE, USA) was used to generate immunofluorescent signals, using TSA dye 520 (1:100, anti-CDKN2A), dye 570 (1:100, anti-hCD8), and dye 620 (1:100, anti-hCD4). Spectral 4,6-diamidino-2-phenylindole (DAPI) (1:10, Akoya) was used to counterstain the samples. The Vectra^®^ 3.0 Automated Quantitative Pathology Imaging System was used for multi-slide imaging. The image data were extracted using the inForm^®^ Software v2.6 (Akoya).

### Flow cytometry

The tumors were harvested and minced into small pieces, and then digested with collagenase type IV (Sigma, 1 mg/mL) and DNase I (Invitrogen, 20 ug/mL) for 30 min at 37 °C, followed by filtering using a 70 μm cell strainer. The cells were washed with PBS and then suspended in fluorescent-activated cell sorting (FACS) staining buffer (1 PBS with 1% BSA and 0.1% sodium azide) for staining. The BD LSRFortessa instrument (BD Bioscience) was used for data acquisition, while FlowJo V10 (BD Bioscience) was used for data processing.

### Statistical analysis

The data were presented as mean ± standard error, with a *p-*value of < 0.05 denoting a significant difference. The differences between groups were analyzed using one-way ANOVA or t-test. All statistical analyses were performed using Prism 8 and SPSS 23.0. Data visualization was performed in R version 4.1.1 using the ggplot2, ComplexHeatmap, gtrellis, and maftools packages.

## Results

### High quality establishment of a PDX-based biobank for CC

**Fig. 1:**
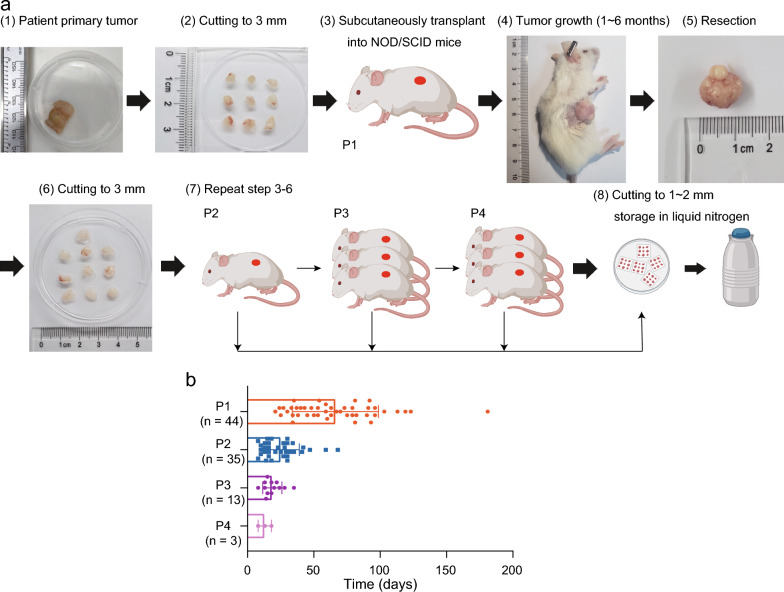
Flowchart of the patient-derived xenograft (PDX) process. **a** Generation process of PDX: (1) The patient’s primary tumor was collected and (2) cut into small pieces (3–4 mm), divided into several parts for subsequent (3) transplantation into the flanks of NOD/SCID (subcutaneously). (4) The growth of P1 generation PDX tumor was observed and recorded. (5) When the PDX tumor grew to 1500 mm^3^ in size, its growth time was recorded. The PDX tumor was obtained, and (6) was cut again to 3 mm in size (7) for the subsequent establishment of P2 to P4 generations PDX tumors. (8) Finally, PDX tumors of different generations were cut and frozen for sequencing or other experiments. **b** The growth time of different generations of PDX tumor (P1–P4). P1 (n = 44; range, 21–181 [mean = 65] days), P2 (n = 35; range, 8–47 [mean = 25] days), P3 (n = 13; range, 8–35 [mean = 18] days), and P4 (n = 3; range, 8–18 [mean = 13] days)

To establish a PDX-based biobank for CC, fresh tumor samples were extracted from 69 CC patients admitted in Tongji Hospital between June 2018 and January 2021, and the clinicopathological characteristics of these patients are summarized in Table 1. The tumor samples were cut into 3–4 mm^3^ in size and implanted into the NOD/SCID mice. After a period of engrafting growth, 44 of the 69 tumors formed PDX xenografts successfully. The success rate of our xenograft model is 63.8%, which is consistent with the 48–75% rate reported by others [31–33]. In order to obtain stable transplanted tumors, some PDX tissues (designated as P1 generation) obtained from primary tumor tissue transplantation were reimplanted into NOD/SCID mice to obtain P2-P4 generation transplanted tumors (Fig. 1a). Interestingly, it took 1 to 6 months for most P1 generation tumors to grow to palpable after implantation, with an average time of 2 months, but the growth time to palpable decreased significantly with the increase of PDX generation (Fig. 1b), indicating the higher adaptation of human tumors to the local environment of immunodeficient mice at later generations.

**Table 1:** The clinicopathological characteristics of samples used for establishing CC PDXs

Variable	All patients	Engrafters	Non-engrafters	Take rate (%)	*p*
	(n = 69)	(n = 44)	(n = 25)		
Median age at surgery, years (IQR)	52 (47–58)	51 (45–56)	55 (50, 60)		0.907
Histology, n (%)					0.865
SC	56 (81.1)	35 (79.6)	21 (84.0)	62.5	
AC	9 (13.1)	6 (13.6)	3 (12.0)	66.7	
Others	4 (5.8)	3 (6.8)	1 (4.0)	75	
Tumor stage^a^, n (%)					0.275
Early	21 (30.4)	12 (27.3)	9 (36.0)	57.1	
LACC	28 (40.6)	17 (38.6)	11 (44.0)	60.7	
Advanced	20 (29.0)	15 (34.1)	5 (20.0)	75	
Tumor differentiation, n (%)					0.915
Poor	34 (49.3)	22 (50.0)	12 (48.0)	64.7	
Moderate	33 (47.8)	21 (47.7)	12 (48.0)	63.6	
Well	2 (2.9)	1 (2.3)	1 (4.0)	50	
Tumor size, n (%)					**0.021**
≥ 4 cm	46 (66.7)	25 (56.8)	21 (84.0)	54.4	
< 4 cm	23 (33.3)	19 (43.2)	4 (16.0)	82.6	
Tumor invasion, n (%)					0.831
Superficial	21 (30.4)	13 (29.5)	8 (32.0)	61.9	
Deep	48 (69.6)	31 (70.5)	17 (68.0)	64.6	
LN metastases, n (%)					0.215
Yes	20 (29.0)	15 (34.1)	5 (20.0)	75	
No	49 (71.0)	29 (65.9)	20 (80.0)	59.2	
LVSI, n (%)					0.109
Yes	28 (40.6)	21 (47.1)	7 (28.0)	75	
No	41 (59.6)	23 (52.9)	18 (72.0)	56.1	
SMI, n (%)					0.583
Yes	9 (13.1)	5 (11.4)	4 (16.0)	55.6	
No	60 (86.9)	39 (88.6)	21 (84.0)	65	
NI, n (%)					0.097
Yes	5 (7.3)	5 (11.4)	0 (0.0)	100	
No	64 (92.7)	39 (88.6)	25 (100.0)	60.9	
PI, n (%)					0.63
Yes	4 (5.8)	3 (6.9)	1 (4.0)	75	
No	65 (94.2)	41 (93.1)	24 (96.0)	63.1	
Prognosis, n (%)					0.236
Non-recurrence	57 (82.6)	34 (77.3)	23 (92.0)	59.7	
Recurrence	1 (1.4)	1 (2.3)	0 (0.0)	100	
Death	2 (2.9)	2 (4.5)	0 (0.0)	100	
Unknown	9 (13.1)	7 (15.9)	2 (8.0)	77.8	
NACT, n (%)					0.63
Yes	4 (5.8)	3 (6.9)	1 (4.0)	75	
No	65 (94.2)	41 (93.1)	24 (96.0)	63.1	

AC adenocarcinoma, LN lymph node, LVSI lymphatic vascular space invasion, NI nerve invasion, NACT neoadjuvant chemotherapy, PI parametrial invasion, SC squamous carcinoma, SMI surgical margin involvement

a Diagnosis was made according to the 2018 staging system of the International Federation of Gynecology and Obstetrics

### CC PDX model maintains the histological and genomic features of PTs

To determine whether the established PDX tumors reflected the histological characteristics of the corresponding PTs, H&E staining was performed on 32 PDX/PT tissue pairs. Based on the cellular morphology and architecture of the samples showed by H&E staining, two independent pathologists confirmed that 96.8% (31/32) of the PDX and PT pairs were concordant (Additional file 1: Fig. S1a), indicating that the PDX tumors could accurately reflect the histological features of the PTs.

**Fig. 2:**
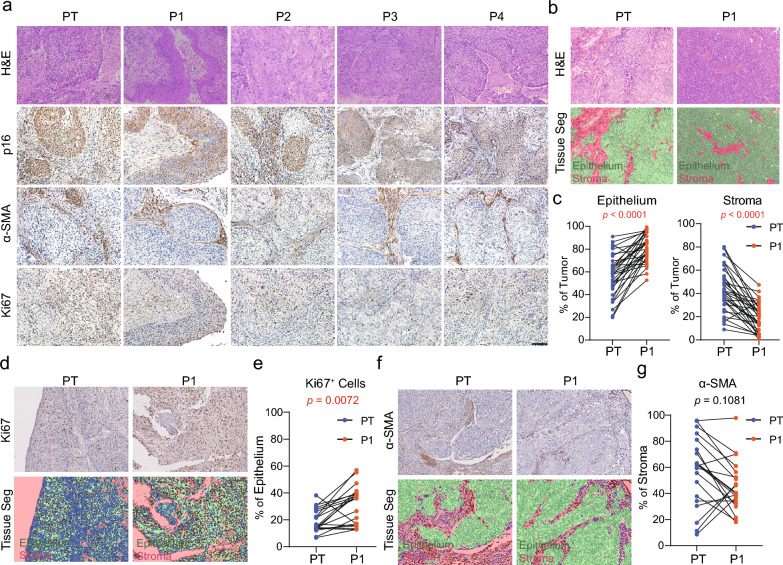
The histological characteristics of primary tumors and matched P1–P4 generations PDX models. **a**. Cervical cancer primary tumor and corresponding PDX models (P1–P4) showed similar histological features as assessed by hematoxylin and eosin and immunohistochemical staining: PDX tumor showed similar levels of α-smooth muscle actin (α-SMA), p16, and Ki67 staining of their parental tumors. **b**, **c** Quantitation of the epithelial and stromal components of the primary tumor and corresponding PDXs (n = 32). **d**, **e**. Quantitation of Ki67 staining in the epithelial fraction (n = 20). **f**, **g**. Quantitation of α-SMA staining in the stromal fraction (n = 20). Scale bar = 50 μm

To assess the concordance at the levels of specific cellular components, P16INK4A staining was used to identify the epithelial cells, α-smooth muscle actin (α-SMA) staining was used to assess the presence of myofibroblasts in the stromal compartment, and Ki67 staining was used to assess tumor cell proliferation. As showed by the example of CAT070, which includes 4 generations of PDX tumors, all of these factors were well-preserved in the P1-P4 PDX tumors (Fig. 2a**)**.

The inForm software was used to score and assess the epithelial and stromal components of 20 pairs of PTs and PDX-P1 tumors. Generally, higher percentage of epithelial and fewer percentage of stromal components were detected in the PDX tumors compared with their PT tissues (Fig. 2b, c and Additional file 1: Fig. S1b). Next, these tumors were compared for the expression of Ki67 and α-SMA in the epithelial and stromal region, respectively. The proliferation rate was increased (Ki-67^+^) in the PDX tumors (Fig. 2d, e), whereas a slight decrease was observed in the α-SMA expression levels of PDX tumors (in the stroma) (Fig. 2f, g).

During implantation and successive passage, human xenografts may undergo genetic alteration with the purpose of adapting to the new in vivo environment, thus raising concerns about the stability and reliability of PDX models [34–36]. To determine whether our established PDX tumors could accurately reflect the genomic features of their corresponding PTs, WES was performed on 10 paired PTs and PDX tumors to compare their genetic differences. We initially examined the tumor purity and ploidy using the absolute algorithm (Additional file 1: Fig. S2a). Most PDX samples at P1 generation demonstrated unchanged or even increased tumor purity compared with their corresponding PTs. Tumor purity was also maintained in the later generation tumors (P2, P3 and P4 generations). For example, the tumor purity of CAT082 at P2 generation was 0.84, compared with 0.78 at P1 and 0.58 in PT. CAT061 PT, which showed the highest purity among the all PTs analyzed (0.96), retained its high tumor purity at the later generations, except for P3 generation, which displayed a tumor purity of 0.49. In addition, the ploidy profile of xenografts matched with their original PTs in all samples analyzed. Therefore, the successfully established PDX tumors highly preserved the tumor purity and ploidy of their corresponding PTs, therefore enabling the comparisons of other genomic features.

**Fig. 3:**
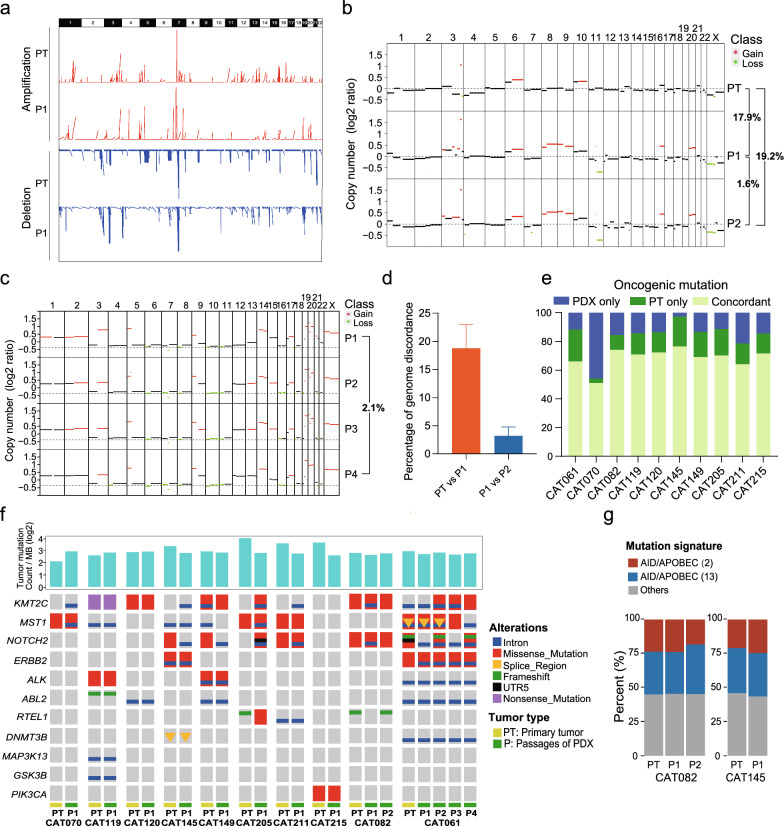
The genomic characteristics of primary tumors and matched P1-P4 generations PDX models. **a** Genome-wide amplifications and deletions in cervical cancer PDX tumors (10 pairs) **b** Copy number (CN) differences among primary tumor (PT), matched P1 and P2 PDX tumors of patient CAT082. Red, CN gain; Green, CN loss. **c** CN differences among PT, matched P1 and P4 PDX tumors of patient CAT061. Red, CN gain; green, CN loss. **d** The percentage of discordant CN alternation of genome between PT and P1 tumor, or between P1 and P2 tumor. **e** The oncogenic mutation status of selected genes in primary tumors and paired PDX models that are successfully engrafted in mice (n = 10). **f** The tumor mutation burden and the alteration status of selected cancer-associated genes in primary tumors and paired PDX tumors. **g** AID/APOBEC mutational signatures (signatures 2 and 13) in CAT082 and CAT145

Next, we assessed the CNVs using GISTIC analysis [29]. A representative picture comparing 10 paired PDX-P1 tumors and PTs for the genomic distribution of CNV amplification and deletion is shown in Fig. 3a, which showed, undoubtedly, similar amplification and deletion profiles between the PDX-P1 tumors and their corresponding PTs. Then, log_2_(CN ratio) was used to quantify the level of CNVs [30] followed by comparing the values between the PTs and paired PDX tumors (P1 to P4). As shown in Fig. 3b, the mean discordant rate between PTs and paired PDX-P1 tumors was 18.79% (range, 3.03–32.80%), indicating the high concordance between PTs and the early generation PDX tumors. Successive passages seem to have minimal influence on CNVs, which is supported by the mean discordant rate of 3.21% (range, 1.65–4.77%) between paired P1 and P2 PDX tumors (Fig. 3d). This notion is further potentiated by the observation that the discordant rate between P1 and P4 tumors was only 2.1% in CAT061 (Fig. 3c).

We further analyzed the somatic mutation profiles of PTs, early (P1) and late (P4) generation tumors after dynamic adjustment. The most frequently mutated genes and oncogenic alterations identified in the PDX tumors and PTs from ten paired samples included KMT2C (75%), MST1 (67%), and NOTCH2 (63%). SNV analysis demonstrated high concordance (70.3%) among PTs, early and late generation PDX tumors (Fig. 3e, f, Additional file 1: Fig. S2b). The mutational burden (TMB) of tumor genomics was generally low, with < 4 mutations per Mb observed across the patient’s tumors and the corresponding PDX models (Fig. 3f). Thus, our PDX model produced a remarkably similar profile to its corresponding PT with minor dynamic modifications of the abnormalities.

Furthermore, the activation-induced cytidine deaminase (AID)/apolipoprotein B editing complex (APOBEC) family has been shown associated with mutational signatures 2 and 13, which was frequently observed in cervical squamous carcinoma [37]. As shown in Fig. 3g and Additional file 1: Fig. S2c, a similar AID/APOBEC mutation signature was noted in CAT145 and CAT082, indicating that characteristic mutational signatures for CC were also maintained in our PDX tumors.

### Establishment of PDX model is influenced by the clinical characteristics, immunological features, and transcriptomics profiles of PTs

Although our PDX model was established at a respectable success rate, there remained 25 unsuccessful attempts notwithstanding the application of standard procedures. Therefore, we evaluated whether PT- or patient-intrinsic factors were responsible for the differential outcomes of implanted xenografts. Our initial analysis examined the clinicopathological parameters of PTs to determine their influence on xenograft engraftment. We found that PTs with the largest diameter ≥ 4 cm had a significantly higher success rate than those < 4 cm, indicating that a larger PT size might promote xenograft engraftment. With regard to tumor stage, although the success rate was numerically higher with the advanced tumor stage, the difference was not significant (Table 1). Interestingly, five tumors obtained from patients with nerve invasion were all successfully engrafted into the NOD/SCID mice, suggesting that tumors with nerve invasion may be another promoting factor for xenograft implantation (Table 1).

**Fig. 4:**
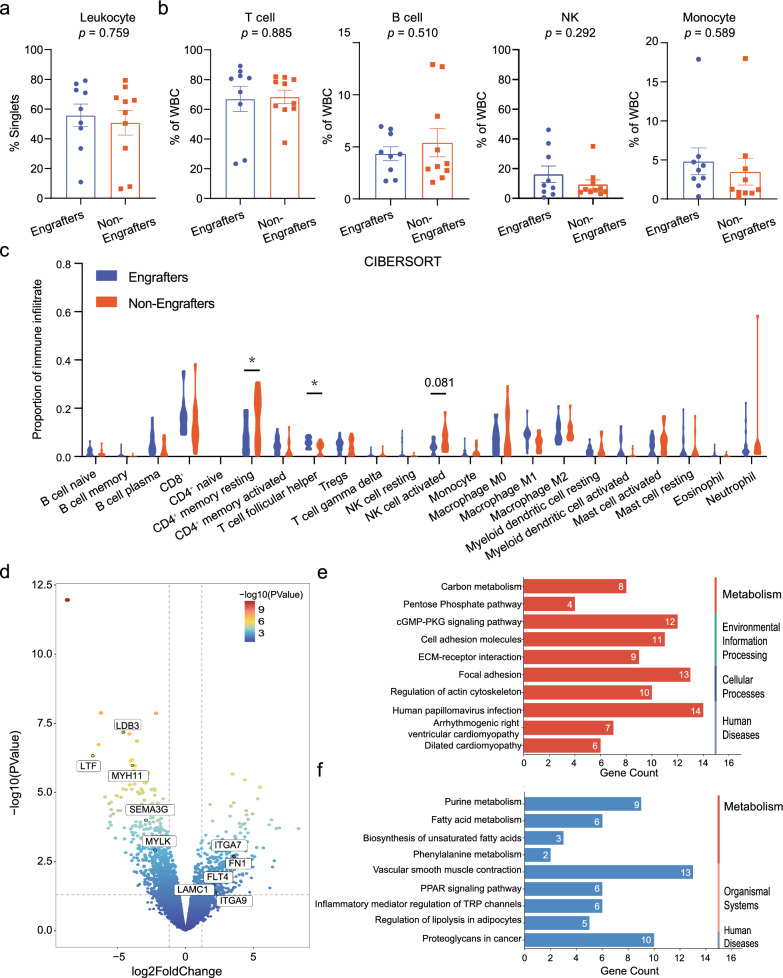
The overview of tumor immune microenvironment and transcriptome profiles of Engrafters and Non-Engrafters. **a** The proportion of leukocytes in Engrafters and Non-Engrafters. **b** The proportion of T, B, and natural killer cells and monocytes in Engrafters and Non-Engrafters. **c** The proportion of immune cell subsets in Engrafters and Non-Engrafters according to the Cell-type Identification by Estimating Relative Subsets of RNA Transcripts. **d** Volcano plots illustrating the differentially expressed genes (DEGs) of primary tumors that were successfully engrafted (n = 16) versus those that were not successfully engrafted (n = 10). In total, 564 DEGs (258 upregulated and 306 downregulated) were founded. **e**, **f** The gene set enrichment analysis for engrafters versus non-engrafters (blue, downregulated pathways; red, upregulated pathways). The data are represented as the mean ± standard error of the mean. Statistical analyses are performed using the Mann–Whitney U test. ^*^p < 0.05

To investigate whether the immune infiltration of PTs influences xenograft engraftment, we compared the immunological characteristics of nine PTs and PDX tumor pairs (engrafters) and ten PTs with its unsuccessful engraftment attempts (non-engrafters) using flow cytometry analysis. The representative gating strategies for immune cell populations and their functional status were shown in Additional file 1: Fig. S3a. No significant difference was found between engrafters and non-engrafters in terms of the relative frequencies of major immune cell subsets including the total number of leukocytes, T cells, B cells, natural killer (NK) cells, and monocytes (Fig. 4a, b), as well as some functional subpopulations of T cells (Additional file 1: Fig. S3b–d). Since the limited availability of single cell suspension prevented us from comparing more immune cell subpopulations in flow cytometry analysis, RNA sequencing and bioinformatics analysis were next performed on 16 and 10 PT samples from the engrafter and non-engrafter groups, respectively. Deconvolution of RNA sequencing data using the Cell-type Identification by Estimating Relative Subsets of RNA Transcripts (CIBERSORT) algorithm enabled the determination of more detailed immune cell subsets, which showed that the engrafters had an increased number of CD4^+^ follicular helper (Tfh) cells and a decreased number of resting CD4^+^ memory and activated NK cells when compared with the non-engrafters. The frequencies of other cell subtypes were comparable between the two groups (Fig. 4c). Thus, our measurements at both transcriptional and protein levels suggest that certain tumor-infiltrating immune cells, particularly Tfh, resting CD4^+^ memory, and activated NK cells, could be key contributors to the outcomes of implanted xenografts.

Subsequently, we analyzed the differential expressed genes (DEGs) between engrafters and non-engrafters and identified 564 DEGs (*p* < 0.05 and logFc > 2), with 258 up-regulated and 306 down-regulated in engrafters compared with non-engrafters (Fig. 4d). Based on KEGG enrichment analysis, the up-regulated DEGs in the engrafters group were enriched in pathways related to cell attachment: cell adhesion pathway and ECM-receptor interaction pathway. These findings suggest that cell attachment may facilitate xenograft engraftment. By contrast, *MYH11* and *MYLK* in the “vascular smooth muscle contraction” pathway were up-regulated in the non-engrafters group, indicating that the stromal component from the patients may contribute to engraftment failure (Fig. 4e, f).

The PT tissues with a faster rate to xenograft establishment (rapid-engrafters) is associated with poor survival outcome in patients with human papillomavirus infection-negative head and neck squamous cell carcinoma [38]. In our cohort, we observed variations in the engrafting speed of PT tissue to PDX-P1 tumors from different patients, which ranged from 21 to 181 days with an average of 65 days. To investigate the relationship between engrafting speed and clinical outcome in our cohort, we used 65 days as the cutoff value to classify rapid-engrafters (n = 25) and slow-engrafters (n = 18). We compared the clinical outcomes and clinicopathological features between the two groups, rapid-engrafters had higher proportion of lymph node (LN) metastasis and large tumors (tumor size ≥ 4 cm) (Additional file 1: Table. S3). To further explore the relationship between engrafting speed and clinicopathological features, we combined the slow-engrafters and non-engrafters into a new group (n = 44). The slow/non-engrafters had significantly low proportion of patients with LN metastasis and large tumors compared with the rapid-engrafters (Table 2). This result suggests that larger tumor or tumors with LN metastasis may be more likely to form xenografts in immunocompromised mice at a higher speed.

**Table 2:** The clinicopathological characteristics of CC patients with rapid-engraftment (n = 25) and slow- and non-engraftment (n = 44)

Variable	Rapid-engrafters (n = 25)	Slow- and non- engrafters (n = 44)	*p*
Median age at surgery, years (IQR)	48	54	0.345
	(43–54)	(48–59)	
Histology, n (%)			0.865
SC	21 (84.0)	35 (79.6)	
AC	3 (12.0)	6 (13.6)	
Others	1 (4.0)	3 (6.8)	
Tumor Stage^a^, n (%)			0.057
Early	4 (16.0)	17 (38.6)	
LACC	10 (40.0)	18 (40.9)	
Advanced	11 (44.0)	9 (20.5)	
Tumor differentiation, n (%)			0.386
Poor	11 (44.0)	23 (52.3)	
Moderate	14 (56.0)	19 (43.2)	
Well	0 (0.0)	2 (4.5)	
Tumor Size, n (%)			**0.001**
≥ 4 cm	10 (40.0)	36 (81.8)	
< 4 cm	15 (60.0)	8 (18.2)	
Tumor Invasion, n (%)			0.449
Superficial	9 (36.0)	12 (27.3)	
Deep	16 (64.0)	32 (72.7)	
LN metastases, n (%)			**0.009**
Yes	12 (48.0)	8 (18.2)	
No	13 (52.0)	36 (81.8)	
LVSI, n (%)			0.663
Yes	11 (44.0)	17 (38.6)	
No	14 (56.0)	27 (61.4)	
SMI, n (%)			0.846
Yes	3 (12.0)	6 (13.6)	
No	22 (88.0)	38 (86.4)	
NI, n (%)			0.433
Yes	1 (4.0)	4 (9.1)	
No	24 (96.0)	40 (90.9)	
PI, n (%)			0.097
Yes	3 (12.0)	1 (2.3)	
No	22 (88.0)	43 (97.7)	
Prognosis, n (%)			0.17
Non-recurrence	20 (80.0)	37 (84.1)	
Recurrence	0 (0)	1 (2.3)	
Death	2 (8.0)	0 (0.0)	
Unknown	3 (12.0)	6 (13.6)	

A Diagnosis was made according to the 2018 staging system of the International Federation of Gynecology and Obstetrics

AC adenocarcinoma, LN lymph node, LVSI lymphatic vascular space invasion, NI nerve invasion, NACT neoadjuvant chemotherapy, PI parametrial invasion, SC squamous carcinoma, SMI surgical margin involvement

We next compared rapid-engrafters and slow/non-engrafters for their differences in immune infiltration and transcriptome profile in the PTs. Our flow cytometric analysis revealed no significant difference in the numbers of major immune cells and T-cell subsets that infiltrated the PTs between the two groups (Additional file 1: Fig. S4a–b). However, the CIBERSORT analysis of transcriptome data showed a significant increase in the expression of CD4^+^ Tfh cells in rapid-engrafters (n = 10) compared with that in slow/non-engrafters (n = 16) (Additional file 1: Fig. S4c). This difference is consistent with that observed in the engraftment and non-engraftment comparison. Additionally, DEG analysis revealed that the expression levels of 114 genes were upregulated and 329 genes were downregulated in rapid engrafters compared with that in slow/non-engrafters (*p* < 0.05) (Additional file 1: Fig. S5a). Moreover, the Gene Ontology enrichment analysis showed that the expression levels of genes associated with cell adhesion and focal adhesion were upregulated in rapid-engrafters (Additional file 1: Fig. S5b).

Taken together, our results suggest that the engraftment of xenograft and engrafting speed, to some extent, are influenced by the clinicopathological characteristics, immunological features, and transcriptomic profiles of PTs.

### Utilization of established PDX models for evaluating patient’s response to chemotherapy in CC

We sought to determine whether the aforementioned PDX model could be employed to evaluate the patient’s responses to different treatment modalities in the real-world settings. Specifically, we investigated the efficiency of cisplatin, a commonly used platinum-based chemotherapy drug, on PDX tumors derived from two patients who had shown different response to prior chemotherapy treatment.

**Fig. 5:**
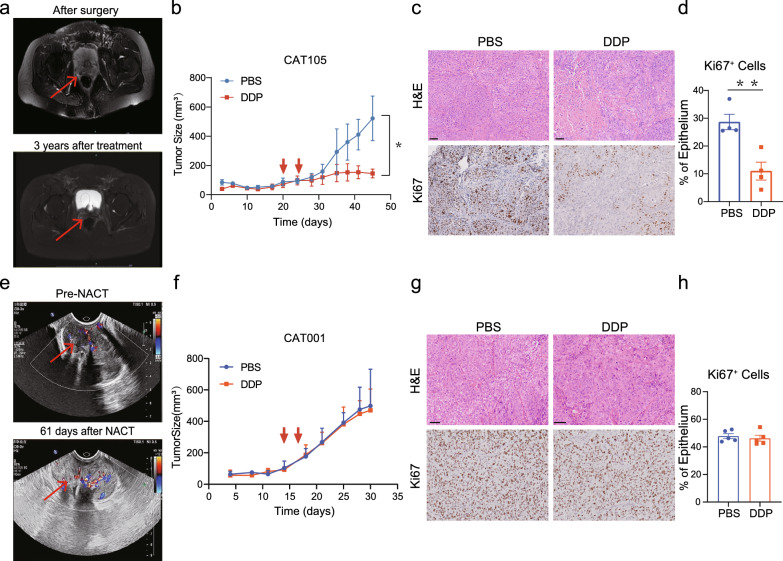
Utilization of established CC PDX models for evaluating patient’s response to chemotherapy. **a**, **e** Imaging of cervical cancer lesions in the patient pre- and post-treatment. **b**, **f** The volumes of PBS and DDP treated PDX tumors. **c**, **g** Representative images of hematoxylin and eosin-stained section and Ki67 staining of PBS and DDP treated PDX tumors; scale bar, 50 μm. **d**, **h** Quantitation of the Ki67 staining in the epithelial fraction (CAT105, n = 4; CAT001, n = 5). ^*^p < 0.05, ^**^p < 0.01, NS, not significant

Patient CAT105 was diagnosed as stage IIA2 locally advanced cervical cancer according to the 2018 FIGO staging system. This patient underwent radical hysterectomy and postoperative pathological examination indicated lymphovascular invasion. Following surgery, this patient received one cycle of docetaxel/cisplatin (DP) treatment and concurrent chemoradiotherapy. No recurrence was observed during the 3-year follow-up period as shown on pelvic magnetic resonance imaging and chest computed tomography (Fig. 5a, Additional file 1: Fig. S6a), indicating that this patient was sensitive to chemoradiotherapy. We implanted the fresh tumor specimen excised from surgery into the NOD-SCID mice, and successful tumor engraftment was achieved. Unsurprisingly, the PDX model retained the pathological characteristics of its corresponding PT, including the histological features of moderately differentiated, as well as the expression of Ki67, p16, and α-SMA (Additional file 1: Fig. S6b).

To assess the sensitivity of PDX model of CAT105 to chemotherapy, phosphate-buffered saline (PBS) or 5 mg/kg of DDP were administered to mice when the tumor size reached to 70 mm^3^. Tumor growth was significantly inhibited in DDP-treated mice compared with that in PBS-treated control mice (*p* = 0.0259), indicating that the xenograft, similar to the primary tumor, is also sensitive to DDP treatment (Fig. 5b). Moreover, the number of proliferating cells was reduced in DDP-treated mice, based on the results of Ki67 staining (Fig. 5c, d). Thus, the PDX model’s sensitivity to chemotherapy alone was in concordance with the patient’s response to chemotherapy in real-world clinical settings.

Subsequently, we investigated whether the PDX model could recapitulated patient’s chemotherapy resistance. In particular, we examined patient CAT001, who had been diagnosed with stage IB2 and had received two cycles of neoadjuvant chemotherapy using cisplatin/paclitaxel. Following treatment, ultrasonography follow-up indicated that the tumor remained stable according to the Response Evaluation Criteria in Solid Tumors (Fig. 5e, Additional file 1: Fig. S6c). The PDX model was derived from untreated specimens, and H&E staining revealed that the PDX model had similar histological characteristics with the PT (Additional file 1: Fig. S6d). In DDP-treated mice, no significant difference was observed in the tumor size when compared with that in the PBS control group (*p* = 0.7551) (Fig. 5f). Moreover, the number of proliferating cells, as determined by Ki67 staining, was almost identical between DDP-treated and PBS groups (Fig. 5g, h). These findings suggest that the PDX model could recapitulate PT resistance to chemotherapy, thus demonstrating its potential as a reliable predictor for patient treatment response.

### Developing combinatorial therapy strategy for HER2-mutant CC using the PDX model

**Fig. 6:**
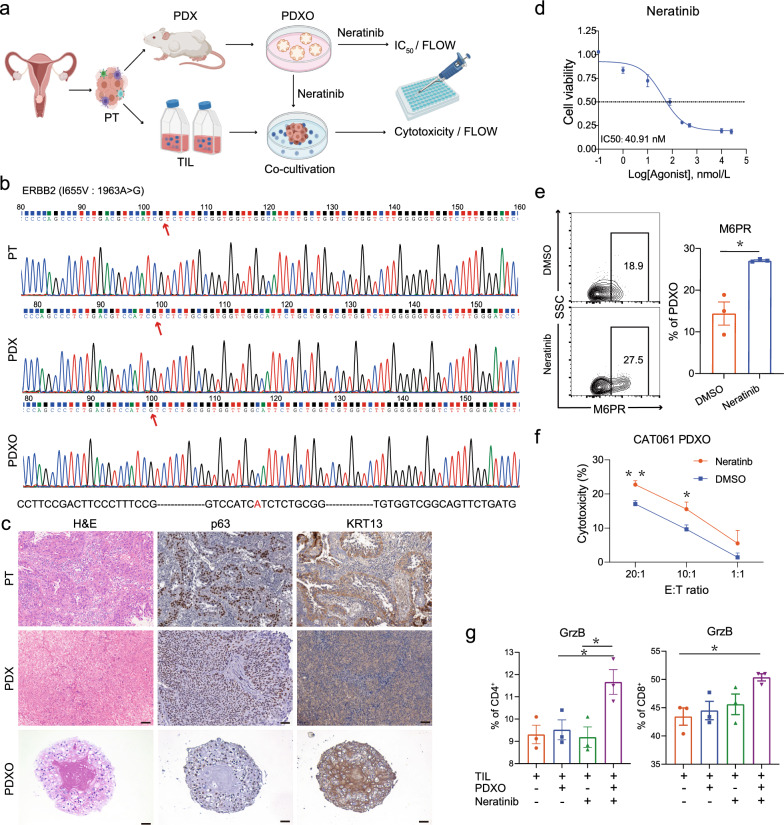
In vitro assessment of human epidermal growth factor receptor 2-targeted and adaptive tumor-infiltrating lymphocyte therapy.** a** Patient-derived xenograft organoid (PDXO) and autologous T-cell coculture system flowchart. **b** HER2-mutation (I655V: 1963A > G) found in primary tumor, PDX, and PDXO. **c** Histology of PDX and PDXO from patient CAT061 (scale bar, 50 μm). **d** Neratinib sensitivity of PDXO from patient CAT061 was determined. The average half-maximal inhibitory concentration from three separate experiments (n = 3) for CAT061 PDXO was 40.91 nM. **e** CAT061 PDXO and TIL cocultures were treated with DMSO or neratinib for 24 h. Mannose-6-phosphate receptor on the surface of CAT061 PDXO tumor cells was determined by flow cytometry. (Student’s t-test, *p* = 0.0102) **f** Cytotoxicity against PDXO cells was measured in a standard 4-h lactate dehydrogenase release assay. **g** Granzyme B expression in CD4^+^ and CD8^+^ T cells of the PDXO and TIL cocultures was determined by flow cytometry (two-way analysis of variance). ^*^p < 0.05, ^**^p < 0.01, NS, not significant

Somatic alterations in HER2 are oncogenic drivers in various cancer types, including CC [17]. Accordingly, neratinib, an irreversible pan-HER2 inhibitor, has shown some promise as a treatment option for advanced-stage CC patients with HER2 mutations [18]. However, the therapeutic efficacy of neratinib remains to be improved. Therefore, in the quest to find a possible cure for CC patients with HER2 mutation, we used the established PDX tumors to explore and evaluate possible treatment options for patients with HER2 mutations. For this purpose, patient CAT061 with stage IIA2 tumor and harboring *HER2* mutations including I655V and P1170A, was selected and the 4 generations of PDX tumor were successfully established. To determine the optimal neratinib concentration, a PDX derived organoid (PDXO) model was established using tumor tissue from the early generation of PDX (Fig. 6a). Both PDX and PDXO tumors preserved the two sites of *HER2* mutations and histological features of their corresponding PT **(**Fig. 6b, c, Additional file 1: Fig. S7a).

We first examined the sensitivity of PDXO to neratinib by culturing PDXO with different concentrations of neratinib for 48 h, through which a half-maximal inhibitory concentration (IC_50_) of 40.91 nM was obtained (Fig. 6d). Interestingly, neratinib-treated PDXO significantly increased the cell surface expression of mannose-6-phosphate receptor (M6PR) compared to DMSO-treated control, as determined by flow cytometry analysis (Fig. 6e). M6PR is a receptor for granzyme B (GrzB) released by activated cytotoxic T cells, and the binding of M6PR to GrzB leads to tumor cell death [39]. Thus, this result suggests that neratinib-treated CC tumor may be sensitive to T cell-mediated cytotoxicity. Several factors have been reported to impact the efficiency of TIL-ACT therapy, such as TIL proportion, tumor mutational burden, viral infection positive and PD-L1 expression [40, 41]. In the tumor tissue of CAT061, we observed a high infiltration of CD3^+^ T cells, which accounted for 38% of the total infiltrated immune cells (Additional file 1: Fig. S7b). Based on the HPV positive status and the high infiltration of CD3^+^ T cells, patient CAT061 may respond well to adoptive T cell therapy, a strategy relied heavily on in vivo model for its evaluation. Thus, a novel TIL/neratinib combinatorial strategy was considered for the treatment of PDX tumor model of CAT061. To this end, we generated TIL products of CAT061 through culturing of the fresh tumor specimens as described in materials and methods. Of note, the TIL products comprised of 81.4% CD4^+^ T cells and 11.4% CD8^+^ T cells (Additional file 1: Fig. S7c), and were almost all in a CCR7^−^CD45RA^−^ effector memory (T_EM_) differentiation status with substantial PD-1 expression. Unsurprisingly, co-culture of TILs and PDXO resulted in detectable cell death of PDXO, and interestingly, PDXO death was further increased upon the addition of neratinib to the culture system (Fig. 6f), indicating a synergistic effect of neratinib combined with effector TILs for the treatment of CC tumor cells. Furthermore, increased expression of GrzB in CD4^+^ and CD8^+^ T cells was observed in the co-culture system containing neratinib (Fig. 6g). In together with the elevated M6PR expression induced by neratinib described in Fig. 6e, our in vitro co-culture experiments suggest that the synergistic effect of TILs and neratinib may be attributable, at least in part, to the M6PR-GrzB interaction.

**Fig. 7:**
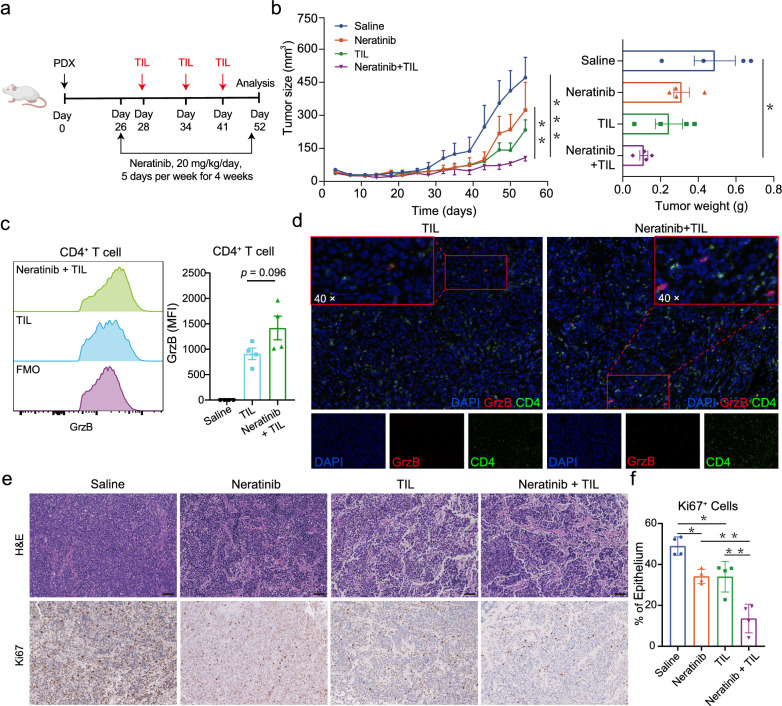
In vivo assessment of HER2-targeted and adaptive tumor-infiltrating lymphocyte (TIL) therapy strategies. **a** PDX tumors of patient CAT061 were treated with neratinib and TIL combination strategy, neratinib or TIL. **b** PDX tumor size of these mice was shown (left graph), and the weights of PDX tumors (day 28) were shown (right graph). **c** Granzyme B (GrzB) expression in CD4^+^ T cells was determined by flow cytometry (Student’s t-test). **d** Multiplex immunofluorescent images of CD4 and GrzB positive cells in the tumor tissue of TIL and TIL combined neratinib treatment groups. **e** Representative hematoxylin and eosin-stained sections and immunohistochemistry images of Ki67 staining of tumors from each of the treatment groups (scale bar, 50 μm). **f** Quantitation of the Ki67 staining in the tumors of the treatment groups. ^*^p < 0.05, ^**^p < 0.01, ^***^p < 0.005, NS, not significant

We next sought to verify the synergistic effects of TILs/neratinib combinatorial therapy in the treatment of in vivo PDX tumor (Fig. 7a). The combination of neratinib and TILs significantly inhibited the PDX tumor growth when compared with the other treatment regimens: vehicle, neratinib alone or TIL alone (Fig. 7b). In addition, the combination of neratinib and TIL did not show toxicity, as none of the treated mice demonstrated weight loss (Additional file 1: Fig. S7d). Furthermore, tumor infiltrating CD4^+^ T cells in the TILs/neratinib group showed higher GrzB expression level than that in the TILs monotherapy group (Fig. 7c), which is further supported by the impressive CD4^+^GrzB^+^ T cells in the microscopy images of TILs/neratinib group (Fig. 7d), despite the comparable T-cell infiltration between the two TIL containing groups (Additional file 1: Fig. S7e). These results were similar to the findings in our in vitro experiments. In addition, Ki67 staining showed that combination treatment also reduced the number of proliferating cells (Fig. 7e, f). Together, these PDX model derived results suggest that the combination of HER2 inhibitors and TILs may be an effective strategy for treating CC patients with HER2 mutations.

## Discussion

Appropriate preclinical models that faithfully reflect the characteristics of the primary tumor can facilitate the development and the efficacy evaluation of novel treatment strategies for solid tumor [9]. In this study, we established, to our knowledge, the largest PDX biobank to date with a 63.8% success rate from 44 patients with different stages of cervical cancer.

PDX models should maintain the genomic features of their parental tumors to serve as a reliable preclinical model for therapeutic drug testing [9, 36]. We compared the difference in genomic alterations between PDX tumors and their matched PTs through WES analyses. Our results indicated that the tumor purity was increased after being engrafted into the mice, which was similar to the findings of other studies [42]. Our analysis showed a 18.79% CNV discordance ratio between PDX-P1 and PT, which is also comparable to the 10–20% ratio in other studies [30]. We obtained a 2.1% CNV discordance ratio between early-generation PDX tumor and late generation tumor, which is, unsurprisingly, similar to the 3%–9% range reported in other studies [30]. In a single-cell genomic analysis of breast cancer, minor subclones dominated the xenografts in consecutive passages, leading to the changes in mutation clusters [43]. We also observed some mutations that were not recorded consecutively in the SNV data, which suggested that clonal dynamics might play an essential role in the tumor evolution of PDX models. Nevertheless, the concordance ratio of SNVs between our PT and PDX models was 70.3%. Overall, our data indicated that the established PDX models retained the genomic mutations and molecular characteristics of the native tumors across serial passaging.

The engraftment rate of PDX model varied widely according to cancer type and individual patient characteristics [44]. In general, CC showed a higher tumor engraftment rate (> 60%) in immunodeficient mice compared with other solid tumor types such as breast cancer (12.5–31.3%) [45, 46]. The success rate of CC PDX establishment is affected by various factors, such as the transplantation site and the time interval between surgery and xenograft implantation [5]. To eliminate batch processing interference, the procedure was standardized to 6 h in order to eliminate the influence of the time interval between surgery and xenograft implantation. The transplant sites for CC PDX model were mainly subcutaneous, orthotopic, and sub-renal capsule. Although the sub-renal capsule and cervical orthotopic engraftment rates were higher (75 and 71.4%, respectively) than the subcutaneous engraftment rate (48–70%) [31, 33, 47], we used subcutaneous implantation in the current study, which is preferred for easier monitoring of the tumor size, and the engraftment rate of 63.8% is sufficient for preclinical investigation.

Our study also investigated several clinical factors that may affect the success rate of PDX engraftment in CC, including tumor size, perineural invasion (PNI), lymphatic vascular space invasion (LVSI), LN metastases, parametrial invasion (PI), and tumor stage. Among them, only tumor size of > 4 cm was associated with the PDX engraftment rate (*p* < 0.05), which was consistent with the report of previous studies on various cancer types [11, 48–50]. Tumor size is an indicator of tumor burden according to the National Cancer Institute and a significant prognostic indicator of advanced-stage CC [51]. Although other pathologic features such as PNI, LVSI, LN metastases, PI, and tumor stage seem to be related to a higher engraftment rate in our study, and these features have been previously associated with PDX formation and poor clinical outcome in other cancer types [50, 52], we did not observe a significant difference in our study (*p* > 0.05). Hence, further studies with a larger sample size and more comprehensive analysis are warranted to confirm these findings.

We also compared the differences in the TME and genomic expression between engrafters and non-engrafters. To compare immune cell infiltration, we utilized flow cytometry to identify immune cell subsets that may correlate with engraftment; however, no significant difference was identified. Using the CIBERSORT algorithm, we observed an increase in the subset of follicular helper T cells, which correlated with engraftment. This finding is in agreement with a previous study, which demonstrated the association of Tfh-like cells with unfavorable outcomes, such as lymphatic metastasis or distant metastasis [53–56]. Furthermore, we demonstrated that activated NK cell and resting CD4^+^ memory cells were unregulated in the non-engrafters group. Consistent with our results, previous studies also revealed that memory T cell and NK cell correlated with better outcomes in other cancer types [57, 58]. With regard to the correlation between tumor molecular features and tumorigenicity, the engrafters group exhibited an enrichment in molecular adhesion-related pathways, which is consistent with the report of previous studies [59]. Moreover, engrafters group was also enriched in the DNA replication and cell cycle pathways in other study [59]. Together, our results suggest that intrinsic factors of primary tumors, such as immune cell subsets and differentially expressed genes, may be useful in predicting the establishment of PDX models.

Previous retrospective studies have suggested that PDX models can be used to predict drug response and outcomes of patients in various cancer types [9, 59, 60]. Our study demonstrated that the PDX models of CC are a promising tool for conventional drug screening and could provide insight into the development of novel therapy. HER2 mutation is a poor prognostic factor of advanced-stage CC [18]. The therapeutic activity of neratinib, a HER2 inhibitor, has been shown in patients with HER2 mutations who were unresponsive to platinum-based chemotherapy (ORR: 25%; 95% confidence interval: 5.5–57.2%) [18]. A previous clinical trial demonstrated that tyrosine kinase inhibitors (TKIs) increased the infiltration of T cells in tumor tissue [19]. In the NeoALTTO trial, higher levels of TILs were associated with improved outcomes in HER2-positive patients treated with lapatinib and/or trastuzumab [20]. Despite the synergistic effect of TILs and HER2 inhibitors suggested by aforementioned investigations, combinatorial strategy using these two treatments has never been explored in CC. Taking advantage of our PDX model, we were able to show that the novel TIL/neratinib combinatorial strategy, featured by an impressive elevation in the infiltration of GrzB^+^ T cells in PDX tumor, may be a promising therapeutic alternative for patients with HER2 mutation. Our results demonstrated that CD4^+^ T cells were dominant in both transfused TIL cell products and PDX tumors that were effectively controlled by combinative therapy. These results suggest that CD4^+^ T cells may be an important anti-tumor cell population in cervical cancer immunotherapy, and it is likely to play a synergistic effect with neratinib against cervical cancer. Previous preclinical studies have supported that CD4^+^ CAR-T cells in maintaining antitumor responses [61]. Although the exact mechanism of action of CD4^+^ T cells remains unclear, their significant antitumor potential can provide new insights into the development of effective tumor immunotherapy [62, 63].

Based on our studies, the PDX tumor for CC could potentially be used to evaluate the effects of various treatment strategies, such as the efficacy of a variety of chemotherapy drugs, the efficacy of chemotherapy drug combination strategies, the screening of second-line drugs, the efficacy of immunotherapy, etc., which will assist in the selection and evaluation of clinical treatment strategies. More importantly, after classifying cervical cancer patients with different molecular subtypes, the PDX tumor model can be used to explore the best treatment strategy for patients with a specific subtype of cervical cancer, thereby providing more rapid and reliable treatment for cervical cancer patients with the same subtype characteristics. Furthermore, we are using the cervical cancer PDX models to further explore the tumor growth characteristics of cervical cancer patients with different molecular subtypes, and the possible intervention strategies and their efficacy.

However, there are several caveats in PDX models, including ours. First, the stromal content is decreased in PDX tumors compared to their corresponding PTs (Fig. 2c). Previous investigations also indicated that human stromal cells are replaced by mouse counterpart over serial passages. Thus, this model is not fit for studying the crosstalk between human cancer cells and stromal cells, and is incapable of recapitulating the contribution of stromal cells to drug sensitivity/resistance. Second, upon implantation into immunocompromised mice and over consecutive passages, human immune cells are gradually lost due to the lack of human cytokines to support their survival (data not shown). Therefore, PDX model is intrinsically unable to assess the impact of patients’ immune system when used for preclinical evaluation. Nevertheless, humanized mice that harbor reconstituted human immune system could overcome this drawback to some extent. In addition, this model is particularly useful for assessing the therapeutic efficacy of adoptively transferred immune cells including CAR-T, TCR-T and TILs. Indeed, our PDX model is a good fit for evaluating the efficacy of TIL-based monotherapy and combinatorial therapy, as demonstrated by our current study. TIL-based immunotherapy is a highly personalized treatment modality, for which the expansion of autologous TIL requires 4–8 weeks and is therefore time-consuming. However, this time interval can be fully utilized to establish PDX tumors, which could be ready at the time of TIL product collection. In fact, we are designing a co-clinical trial for TIL/neratinib combinatorial therapy in r/m CC, which is similar to our current strategy but in a prospective setting. In this scenario, our PDX model will act as an “avatar” that is able to timely guide clinical decision-making in selecting appropriate therapeutic strategies, i.e., monotherapy with either TIL or neratinib, or TIL/neratinib combinatorial therapy. Based on our current study, this co-clinical trial is promising and will provide insight into future development of novel therapeutic strategies for r/m CC.

## Conclusions

Our study had successfully established a large and diverse PDX biobank that accurately reflects the biological and genomic heterogeneity of patients with CC. Our PDX models can be a valuable tool for predicting the treatment response in individual patients. Furthermore, our study identified key factors that influence PDX engraftment, such as tumor size, immune microenvironment, and cell adhesion pathway-related genes. Finally, using the PDX model, we found that combinatorial therapy with ACT and neratinib could effectively inhibit the growth of PDX tumors derived from CC patients with HER2-mutation. Overall, our study highlights the potential of PDX models as a tool for developing more effective therapy for advanced-stage CC.

### Supplementary Information

**Additional file 1: Fig. S1 **Histological characteristics of primary tumors and paired PDX models.** a**. The histological features of cervical cancer primary tumors and corresponding PDX tumors were assessed by hematoxylin and eosin staining in three patients. **b**. Quantitation of the epithelial and stromal components of the tumor and corresponding PDXs. **Fig. S2 **Genomic characteristics of primary tumor and paired PDX models. **a**. Whole-exome sequencing-based estimates of the purity and tumor ploidy of 10 paired samples. **b**. The percentage of the SNP genome discordance between PT and P1, or between P1and P2. **c**. AID/APOBEC mutational signatures (signatures 2 and 13) in PDXs. **Fig. S3 **Gating strategy of immune cell subsets in the tissues of primary tumors** a**. Gating strategy used for the analysis of leukocyte and T-cell subpopulations. **b**. The proportions of Treg and Trm in the engrafter and non-engrafter groups. **c and d**. The proportions of T_EMRA,_ T_EM,_ T_CM,_ T_N_, and PD-1 positive fraction in CD4^+^ and CD8^+^ T cells in the engrafter (n = 6) and non-engrafter (n = 4) groups. These data are represented as the mean ± standard error. Statistical analyses were performed using the Mann–Whitney U test. **Fig. S4 **Tumor immune microenvironment of rapid engrafters and slow and non-engrafters. **a**. The proportions of T, B, and natural killer cells, monocytes, and T-cell subpopulations. (Rapid engrafters [n = 6] and slow and non-engrafters [n = 13]). **b**. The proportions of T_EMRA,_ T_EM,_ T_CM,_ T_N,_ and PD-1 positive fraction in CD4^+^ and CD8^+^ T cells in rapid-engrafters (n = 4) and slow- and non-engrafters (n = 6) groups. **c**. The proportions of immune cell subsets in the tumor according to the Cell-type Identification by Estimating Relative Subsets of RNA Transcripts. Rapid engrafters (n = 10), slow and non-engrafters (n = 16). These data are represented as the mean ± standard error. Statistical analyses were performed using the Mann–Whitney U test. **Fig. S5 **Transcriptome profiles of rapid and slow and non-engrafters. **a**. Volcano plots illustrating the differentially expressed genes (DEGs) of primary tumors that were successfully engrafted (n = 16) versus those that were not successfully engrafted (n = 10). In total, 564 DEGs (258 upregulated and 306 downregulated) were founded. **b**. The gene set enrichment analysis of rapid engrafters. **Fig. S6 **Pathological characteristics of primary tumor tissue and PDX in two cervical cancer patients with chemotherapy. **a. **Pre-surgery imaging of cervical cancer lesions in patient CAT105. **c. **Imaging of cervical cancer lesions in patient CAT001 at the time of 3 years after treatment. **b and d.** Hematoxylin and eosin staining and α-smooth muscle actin, p16, and Ki67 staining for the primary tumor and paired PDX tumor of patient CAT001 and CAT105 (20× magnification). **Fig. S7** In vitro and in vivo assessment of HER 2-targeted and adaptive tumor-infiltrating lymphocyte (TIL) combinative therapy strategies. **a**. HER2 mutation (P1170A: c.3508C>G) was found in primary tumor, PDX and PDXO. **b**. T-cell populations in the immune cell infiltration of patient CAT001 were determined by flow cytometry. **c**. T-cell subpopulations in the TIL culture products of patient CAT001 were determined by flow cytometry. **d**. The body weight of the mice in different groups. **e**. Multiplex immunofluorescent images of CD4, CD8, and p16 positive cells in the tumor tissue of TIL and TIL combined neratinib treatment groups. **Table S1.** Primary antibodies used in this study. **Table S2.** Chemicals and recombinant proteins used in this study. **Table S3.** The clinicopathological characteristics of CC patients with rapid-engrafment (n = 25) and slow- engrafment (n = 19)

## Data Availability

All data required to evaluate the conclusions in the paper are present in the paper and additional files. The raw sequence data are stored in the National Genomics Data Center (https://ngdc.cncb.ac.cn/) with the accession number CRA009399 and HRA005334. Other data and materials are available from the corresponding authors upon reasonable request.
