# Supplementary material for: Establishment of a high-fidelity patient-derived xenograft model for cervical cancer enables the evaluation of patient’s response to conventional and novel therapies
Source: J Transl Med. 2023 Sep 9;21:611. doi: 10.1186/s12967-023-04444-5 (PMC10492358; doi:10.1186/s12967-023-04444-5)
Supplement: Supplementary file 1 — Additional file 1: Fig. S1 Histological characteristics of primary tumors and paired PDX models. a. The histological features of cervical cancer primary tumors and corresponding PDX tumors were assessed by hematoxylin and eosin staining in three patients. b. Quantitation of the epithelial and stromal components of the tumor and corresponding PDXs. Fig. S2 Genomic characteristics of primary tumor and paired PDX models. a. Whole-exome sequencing-based estimates of the purity and tumor ploidy of 10 paired samples. b. The percentage of the SNP genome discordance between PT and P1, or between P1and P2. c. AID/APOBEC mutational signatures (signatures 2 and 13) in PDXs. Fig. S3 Gating strategy of immune cell subsets in the tissues of primary tumors a. Gating strategy used for the analysis of leukocyte and T-cell subpopulations. b. The proportions of Treg and Trm in the engrafter and non-engrafter groups. c and d. The proportions of TEMRA, TEM, TCM, TN, and PD-1 positive fraction in CD4+ and CD8+ T cells in the engrafter (n = 6) and non-engrafter (n = 4) groups. These data are represented as the mean ± standard error. Statistical analyses were performed using the Mann–Whitney U test. Fig. S4 Tumor immune microenvironment of rapid engrafters and slow and non-engrafters. a. The proportions of T, B, and natural killer cells, monocytes, and T-cell subpopulations. (Rapid engrafters [n = 6] and slow and non-engrafters [n = 13]). b. The proportions of TEMRA, TEM, TCM, TN, and PD-1 positive fraction in CD4+ and CD8+ T cells in rapid-engrafters (n = 4) and slow- and non-engrafters (n = 6) groups. c. The proportions of immune cell subsets in the tumor according to the Cell-type Identification by Estimating Relative Subsets of RNA Transcripts. Rapid engrafters (n = 10), slow and non-engrafters (n = 16). These data are represented as the mean ± standard error. Statistical analyses were performed using the Mann–Whitney U test. Fig. S5 Transcriptome profiles of rapid and slow and non-eng [file 12967_2023_4444_MOESM1_ESM.zip › New folder/SFig 3.pdf]

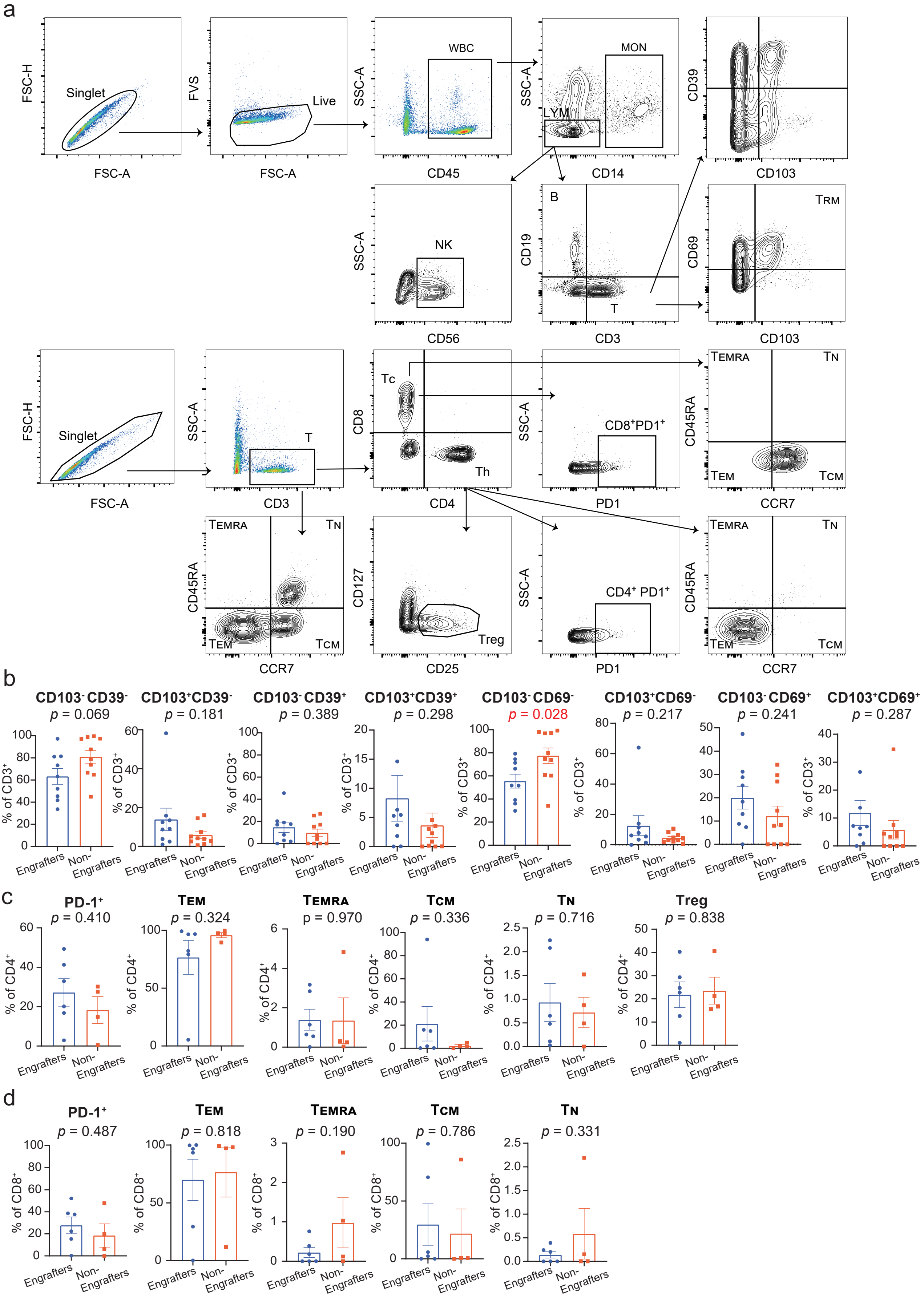

a
FSC-H
Singlet
FSC-A
FVS
Live
FSC-A
SSC-A
WBC
CD45
SSC-A
MON
LYM
CD14
CD39
CD103
SSC-A
NK
CD56
B
CD19
T
CD3
TRM
CD69
CD103
FSC-H
Singlet
FSC-A
SSC-A
T
CD3
Tc
CD8
Th
CD4
SSC-A
CD8+PD1+
PD1
TEMRA
TN
CD45RA
TEM
TCM
CCR7
TEMRA
TN
CD45RA
TEM
TCM
CCR7
CD127
Treg
CD25
SSC-A
CD4+ PD1+
PD1
TEMRA
TN
CD45RA
TEM
TCM
CCR7
b
CD103- CD39-
p = 0.069
CD103+CD39-
p = 0.181
CD103- CD39+
p = 0.389
CD103+CD39+
p = 0.298
CD103- CD69-
p = 0.028
CD103+CD69-
p = 0.217
CD103- CD69+
p = 0.241
CD103+CD69+
p = 0.287
% of CD3+
Engrafters
Non-Engrafters
c
PD-1+
p = 0.410
TEM
p = 0.324
TEMRA
p = 0.970
TCM
p = 0.336
TN
p = 0.716
Treg
p = 0.838
% of CD4+
Engrafters
Non-Engrafters
d
PD-1+
p = 0.487
TEM
p = 0.818
TEMRA
p = 0.190
TCM
p = 0.786
TN
p = 0.331
% of CD8+
Engrafters
Non-Engrafters
